# Effects of virtual reality and layered tooth model training on manual dexterity in preclinical dental education

**DOI:** 10.1186/s12909-025-07622-9

**Published:** 2025-07-07

**Authors:** Esra Yildirim Manav, Sema Yazici Akbiyik, Aydan Büşra Ceylan, Ayfer Ezgi Yilmaz Çakiroğlu, Duygu Tuncer

**Affiliations:** 1 Lokman Hekim Üniversitesi, Ankara, Turkey; 2 Hacettepe University, Ankara, Turkey

**Keywords:** Restorative dentistry, Dental education, Simulator, Virtual reality, Preclinical training

## Abstract

**Background:**

Manual dexterity is a critical component of dental education, especially during the early stages of preclinical training. Recent advancements in simulation technology, such as virtual reality (VR) haptic simulators and layered tooth models such as Caviprep (hereafter referred to as the layered model), offer promising tools to enhance psychomotor skills. This study aimed to explore their combined and individual effects on manual skills development in novice dental students. We hypothesized that combined training with VR and layered models would result in greater improvements in manual skills than either modality alone or conventional methods.

**Methods:**

Forty-eight second-semester dental students were randomly assigned to four groups: Group 1 (the layered model + VR), Group 2 (the layered model only), Group 3 (VR only), and Group 4 (Control; extracted teeth only). All students received identical theoretical instruction and standardized demonstrations for occlusal amalgam cavity preparation. Manual dexterity was assessed using a standardized evaluation form completed by three blinded evaluators. Statistical analyses were conducted using SPSS 23.0, with significance set at *p* < 0.05.

**Results:**

A significant difference was observed among groups in terms of cavity depth accuracy (*p* = 0.001), with the control group (Group 4) performing significantly worse. There were no statistically significant differences in other evaluated parameters including cavity outline, floor smoothness, internal line angles, marginal ridge integrity, retention form, or cavity width (*p* > 0.05).

**Conclusions:**

The integration of VR simulators and layered models enhances depth control during cavity preparation exercises in early dental training. These tools may provide valuable benefits in developing the manual skills essential for clinical competence.

**Trial registration:**

Not applicable.

**Supplementary Information:**

The online version contains supplementary material available at 10.1186/s12909-025-07622-9.

## Introduction

Manual dexterity development is a foundational aspect of preclinical education in restorative dentistry. Dental students must acquire precise motor skills and spatial awareness early in their training to succeed in clinical applications. Traditionally, these skills have been developed through hands-on practice using plastic teeth or extracted human teeth. However, these methods often present limitations, such as inconsistencies in material properties, lack of standardized feedback, and limited opportunities for repetitive and individualized learning experiences [1, 2].

To address these challenges, virtual reality (VR) haptic simulators have been introduced as innovative educational tools in dentistry. These platforms provide interactive and immersive environments that simulate clinical procedures and offer real-time feedback on manual dexterity and performance [3]. Previous studies have shown that VR-based simulators can improve psychomotor skill acquisition, enhance precision in cavity preparation, and increase student confidence by providing a safe and repeatable training experience [4, 5].

In addition to VR technologies, physical simulation tools such as multi-layered training blocks and 3D-printed teeth have been increasingly incorporated into preclinical curricula. These models replicate enamel, dentin, and pulp structures, thereby enhancing spatial perception and offering realistic tactile feedback [6]. For instance, the use of 3D-printed teeth in simulating deep caries has been shown to boost students’ confidence in selective caries removal [7].

Recent innovations in educational technology have highlighted the expanding role of VR simulators in preclinical training. Platforms like the Simodont dental trainer provide highly engaging and clinically relevant environments that promote hands-on skill development [8]. Moreover, anatomically detailed 3D-printed teeth have been found to effectively supplement preclinical education by providing realistic practice scenarios [9].

Despite the growing interest in VR and physical simulation models, the optimal strategies for integrating these technologies into dental education remain under discussion. Some institutions adopt them as essential components of their curricula, while others use them primarily for assessment or remediation. Concerns regarding the validity, reliability, and long-term educational outcomes of these systems still persist [8, 10]. Therefore, evidence-based guidelines are needed to determine the most effective approaches to incorporating simulation technologies into preclinical restorative dentistry education.

This study aimed to evaluate the combined and individual impacts of VR haptic simulators and layered simulation models on manual skill development among second-semester dental students. By comparing different instructional approaches, this research seeks to inform best practices for integrating simulation-based tools into restorative dentistry education. We hypothesized that the combination of VR and layered simulation training would result in superior performance in preclinical manual tasks compared to either method used individually or the traditional training approach alone.

## Materials and methods

### Ethical approval and participants

The study protocol was approved by the Ethics Committee of Lokman Hekim University (Approval No.: 2024 − 279). All second-year dental students enrolled in the preclinical restorative dentistry program were invited to participate. This randomized controlled study was reported in accordance with the CONSORT and STROBE extensions for simulation-based research, as proposed by Cheng et al. [11]. The study’s objectives were explained in detail, and written informed consent was obtained from all participants.

Sample size calculation was performed using G*Power 3.1.9.6. For categorical data, the “Goodness-of-Fit Tests: Contingency Tables” option was used. The power was set at 80% and the alpha level at 0.05. Due to the absence of comparable prior studies, an effect size of 0.50 was assumed. This yielded a minimum required sample size of 44. To account for possible data loss, 48 participants were included. No participants or outcome data were lost during the study. Although the sample size calculation accounted for potential attrition, all 48 participants completed the full protocol.

All extracted mandibular molars used in this study were obtained from the university’s institutional clinical archive with prior ethics committee approval. Informed consent for the use of teeth in research and teaching was obtained at the time of extraction. The teeth were anonymized and stored in 0.1% thymol solution at room temperature until use. Teeth with visible caries, cracks, or restorations were excluded. To ensure standardization, only sound mandibular molars with similar anatomical features were selected. Random allocation of teeth to students was performed to minimize intergroup bias related to tooth morphology.

Inclusion criteria included no previous exposure to VR simulators or the layered model, no experience using an air turbine handpiece, prior completion of the same theoretical restorative curriculum, and basic knowledge of occlusal cavity preparation. Demographic data (age and gender) were collected via Google Forms and anonymized using randomly assigned ID numbers.

### Intervention procedures

All simulation sessions were conducted in the university’s preclinical laboratory using standardized procedures, under the supervision of restorative dentistry faculty. Each simulator was calibrated prior to use to ensure consistency. Initially, all students received a standardized demonstration on occlusal amalgam cavity preparation for mandibular molars, which was performed using typodont teeth mounted on manikins, consistent with the institution’s standard preclinical teaching procedures. A brief lecture was also delivered by restorative dentistry instructors introducing the VR simulator (VirTeaSy Dental©, France) and Caviprep (Edudent, Turkey), hereafter referred to as the layered model. Hands-on demonstrations were conducted using both systems to reinforce learning.

To provide an interactive and equitable learning experience, practical sessions were arranged in small groups of five students.

### Operational protocols for virtual reality and layered simulation models

**Fig. 1:**
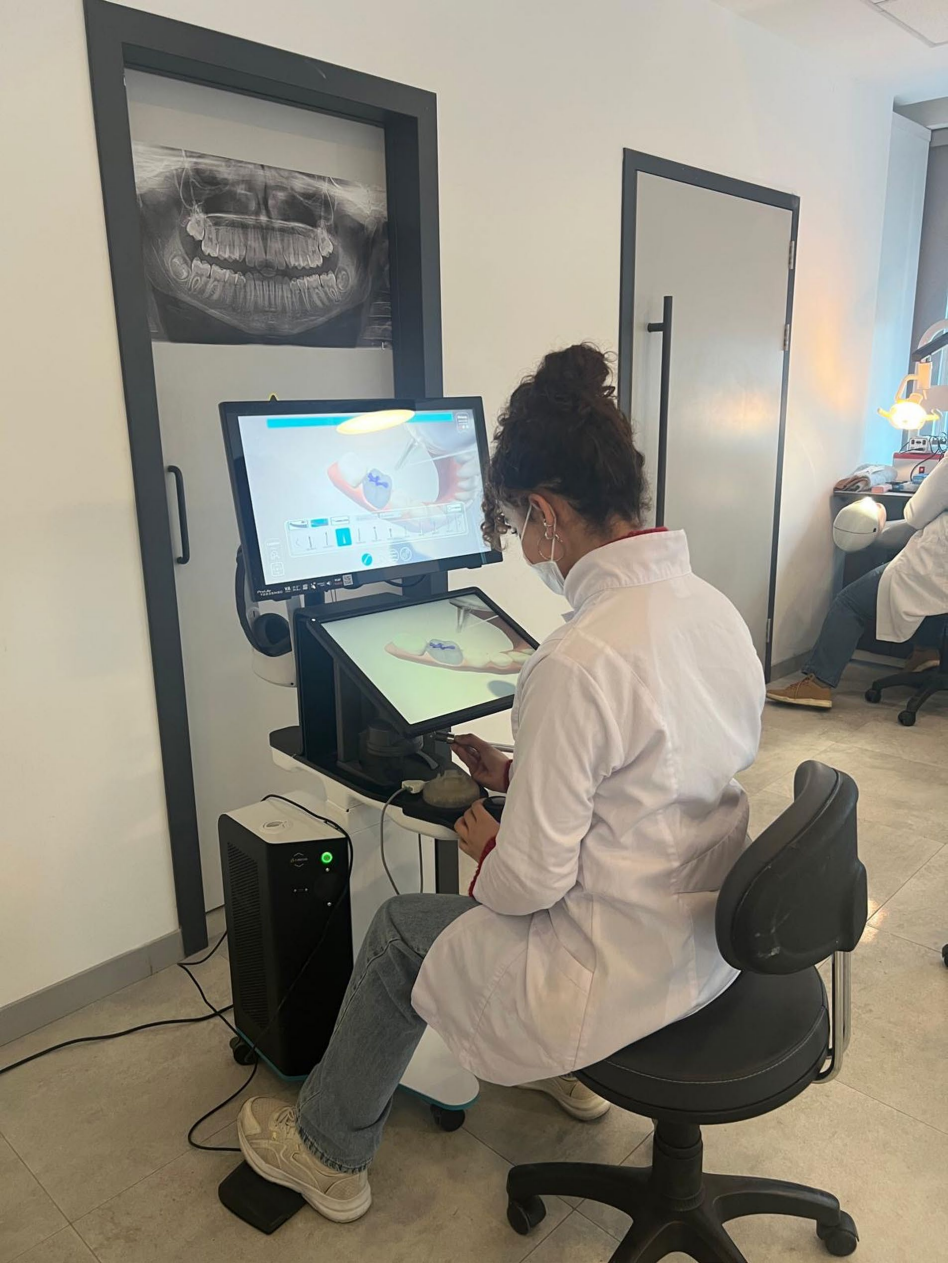
VirTeaSy Dental virtual reality (VR) haptic simulator. The system includes a 3D screen, touchscreen interface, and haptic tools such as a dental mirror and virtual handpiece to provide interactive and immersive operative simulation

The VR haptic simulator and the layered model were used to develop depth control, hand–eye coordination, and spatial awareness in occlusal cavity preparation. The simulation was integrated to enhance psychomotor skill acquisition prior to working on real teeth. The VirTeaSy Dental simulator features a touchscreen and 3D display, offering high-resolution visualization of teeth and instruments. Below the screen are haptic tools, a dental mirror, and a 3D mouse designed for immersive operative simulation (Fig. 1).

**Fig. 2:**
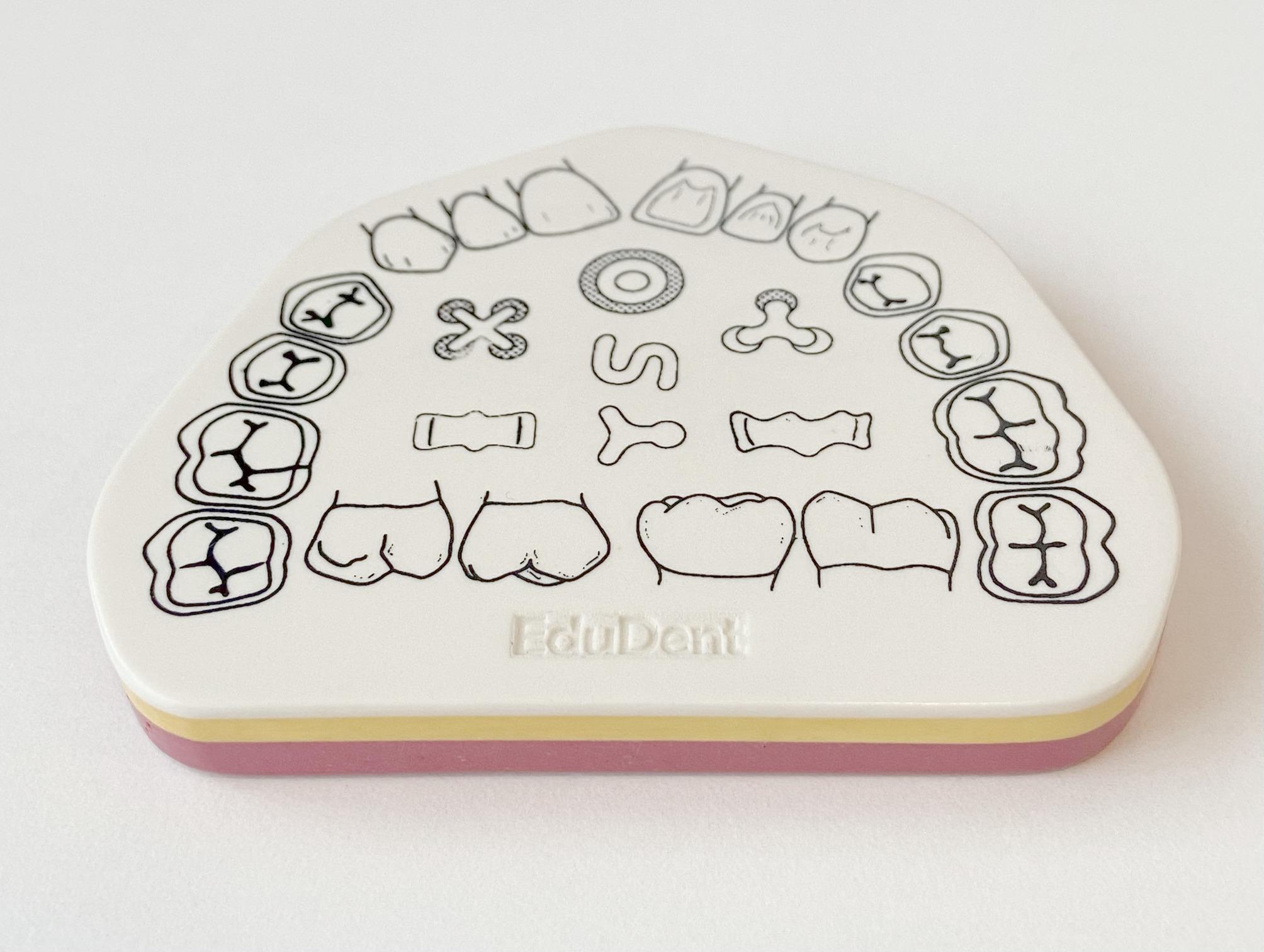
Caviprep layered simulation model used for preclinical training in restorative dentistry. The model features distinguishable layers simulating enamel (white), dentin (yellow), and pulp (red), offering tactile feedback based on varying hardness levels

The layered model replicates enamel, dentin, and pulp with materials of varying hardness. The red layer simulates the pulp and provides distinct tactile feedback, enhancing anatomical awareness (Fig. 2).

### Experimental groups

After the initial demonstration, participants were randomly divided into four groups based on the type of additional training provided. Participants were allocated to groups using simple randomization via a computer-generated random number list (www.random.org), using anonymized student ID numbers to ensure unbiased allocation:

- **Group 1 (The layered model + VR Group**,** n:12)**: Students received hands-on training using both the layered model and the VR haptic simulator.
- **Group 2 (The layered model Group**,** n:12)**: Students received hands-on training using only the layered model.
- **Group 3 (VR Simulator Group**,** n:12)**: Students received hands-on training using only the VR haptic simulator.
- **Group 4 (Control Group**,** n:12)**: Students did not receive any supplementary VR or Caviprep training.

Following training, all students performed occlusal amalgam cavity preparations on extracted mandibular molars.

### Training sessions

Following the initial interventions, students engaged in structured training sessions conducted over two days.

#### Group 1

Students in this group practiced occlusal cavity preparation on mandibular molars using both the VR haptic simulator and the layered model under standardized conditions. Each participant completed three practice sessions with the VR simulator and three sessions with the layered model. After completing the practice sessions, they performed occlusal amalgam cavity preparation on extracted human teeth as part of their preclinical training. No extracted teeth were used during the training phase.

#### Group 2

Students in this group practiced occlusal cavity preparation using only the VR haptic simulator and completed three practice sessions. They did not practice on extracted teeth during the training phase; extracted teeth were used solely for the final assessment.

#### Group 3

Students in this group practiced using only the layered model, completing three cavity preparations. Similar to Groups 1 and 2, extracted human teeth were not used during training but only during the final assessment.

#### Group 4

Students in this group followed the standard institutional protocol by performing occlusal cavity preparation on extracted mandibular molars after attending the theoretical lecture and observing the typodont-based demonstration. Although they did not receive supplementary training with VR or the layered model, they were familiarized with air turbine handpiece use during the procedure and worked under faculty supervision in the preclinical laboratory.

Each training session lasted approximately 20–25 min per student per modality and was conducted under faculty supervision over two consecutive days. The final cavity preparation assessment was performed immediately after the training sessions to minimize variability related to learning retention. During the practice sessions, instructors were present to supervise and respond to technical questions when necessary; however, no direct performance feedback or coaching was provided in order to prevent intervention bias.

To minimize stress and optimize the learning process, no time restrictions were imposed during practice sessions, allowing participants to fully explore and adapt to the features of both the VR simulator and the layered model.

Following the training sessions, all participants completed occlusal amalgam cavity preparation exercises on extracted human teeth during their preclinical laboratory sessions.

### Evaluation of cavity preparations

The quality of the cavity preparations performed on extracted human teeth was evaluated using a standardized assessment form developed in accordance with guidelines established by restorative dentistry specialists (Supplementary Table 1; criteria adapted from [12]). Each parameter was scored based on predefined descriptors in the evaluation rubric. Depending on the criterion, a two-point (0 = unacceptable, 1 = acceptable) or three-point (0 = unacceptable, 1 = acceptable with minor errors, 2 = ideal) scale was used. The complete evaluation form is provided as Supplementary Table 1. All cavity preparations were independently assessed by three blinded evaluators to minimize potential bias. To ensure blinding, each extracted tooth was assigned a randomized anonymized code that concealed the participant’s group. Evaluators did not participate in training sessions and remained blinded to group assignments during scoring. The final preclinical score for each student was determined by calculating the mean of the three evaluators’ scores. These scores were systematically recorded for subsequent statistical analysis, based on expert-defined criteria in line with the learning objectives of the simulation tools. Inter-rater reliability among the three blinded evaluators was calculated using Fleiss’ Kappa, yielding a coefficient of κ = 0.807, which indicates a high level of agreement. Any discrepancies among evaluator scores were resolved through consensus meetings following independent assessments.

### Statistical analysis

The data obtained from this study were analyzed using the Statistical Package for the Social Sciences (SPSS) version 23 and “***irr***” package in R version 4.3.0. The level of agreement among the evaluations by restorative dentistry faculty members was assessed using Fleiss’ kappa coefficient. Differences in cavity preparation evaluations among the four groups (The layered model + VR, The layered model, VR, and Control Group) were examined using Pearson’s chi-square test. In cases where the assumptions for the chi-square test were not satisfied, the Fisher-Freeman-Halton test was applied. The normality assumption was evaluated using the Shapiro–Wilk test, while the homogeneity of variances was assessed with Levene’s test. Differences in overall cavity preparation quality among the groups were analyzed using one-way analysis of variance (ANOVA). Cramér’s V coefficient was reported to measure the effect size for chi-square tests and eta-squared was used for ANOVA [13]. All statistical evaluations were conducted at a significance level of 0.05.

## Results

The comparison of cavity preparation quality among the four training methods (The layered model + VR, The layered model, VR, and Control Group) is presented in Table 1. According to the evaluations conducted by restorative dentistry faculty members, a statistically significant difference was found among the groups in terms of depth of cavity (*p* = 0.001). The Cramér’s V coefficient was found as 0.463, with 6 degrees of freedom(df), indicated a large effect size, suggesting a strong association between the groups in terms of cavity width [13]. No significant differences were observed between the groups regarding the other parameters: cavity outline (*p* = 0.416), floor of cavity (*p* = 0.849), internal line angle (*p* = 0.849), marginal ridge (*p* = 0.352), retention form (*p* = 0.786), and width of cavity (*p* = 0.330).

**Table 1:** Comparison of learning methods based on cavity preparation parameters

Parameter	Score	The layered model + VR	The layered model	VR	Control Group	*p*-value	Cramér’s V
Cavity Outline	0	2/12 (16.7%)	0/12 (0.0%)	3/12 (25.0%)	3/12 (25.0%)	0.416^b^	0.245
	1	6/12 (50.0%)	7/12 (58.3%)	3/12 (25.0%)	6/12 (50.0%)		
	2	4/12 (33.3%)	5/12 (41.7%)	6/12 (50.0%)	3/12 (25.0%)		
Floor of Cavity	0	2/12 (16.7%)	3/12 (25.0%)	3/12 (25.0%)	1/12 (8.3%)	0.849^b^	0.177
	1	10/12 (83.3%)	9/12 (75.0%)	9/12 (75.0%)	11/12 (91.7%)		
Internal Line Angle	0	2/12 (16.7%)	3/12 (25.0%)	3/12 (25.0%)	1/12 (8.3%)	0.849^b^	0.177
	1	10/12 (83.3%)	9/12 (75.0%)	9/12 (75.0%)	11/12 (91.7%)		
Depth of Cavity	0	2/12 (16.7%)	3/12 (25.0%)	2/12 (16.7%)	1/12 (8.3%)	0.001^b*^	0.463
	1	1/12 (8.3%)	3/12 (25.0%)	5/12 (41.7%)	11/12 (91.7%)		
	2	9/12 (75.0%)	6/12 (50.0%)	5/12 (41.7%)	0/12 (0.0%)		
Marginal Ridge	0	3/12 (25.0%)	0/12 (0.0%)	1/12 (8.3%)	0/12 (0.0%)	0.352^b^	0.292
	1	4/12 (33.3%)	4/12 (33.3%)	5/12 (41.7%)	7/12 (58.3%)		
	2	5/12 (41.7%)	8/12 (66.7%)	6/12 (50.0%)	5/12 (41.7%)		
Retention Form	0	5/12 (41.7%)	5/12 (41.7%)	3/12 (25.0%)	5/12 (41.7%)	0.786^b^	0.192
	1	6/12 (50.0%)	6/12 (50.0%)	9/12 (75.0%)	7/12 (58.3%)		
	2	1/12 (8.3%)	1/12 (8.3%)	0/12 (0.0%)	0/12 (0.0%)		
Width of Cavity	0	6/12 (50.0%)	5/12 (41.7%)	9/12 (75.0%)	8/12 (66.7%)	0.330^a^	0.267
	1	6/12 (50.0%)	7/12 (58.3%)	3/12 (25.0%)	4/12 (33.3%)		

a: Pearson’s chi-square test; b: Fisher-Freeman-Halton test results

* indicates statistically significant results (*p* < 0.05)

Further pairwise comparisons for the depth of cavity parameter are summarized in Table 2. The Control Group differed significantly from each of the experimental groups (The layered model + VR, The layered model, and VR) (*p* < 0.001, *p* = 0.001, and *p* = 0.019, respectively). The Cramér’s V coefficients were found to be 0.858 (with df = 2), 0.694 (with df = 2), and 0.562 (with df = 2), indicating large effect sizes and suggesting a strong association between the control group and the other groups in terms of cavity width, respectively. No significant differences were observed between The layered model + VR, The layered model, and VR groups (*p* = 0.524, *p* = 0.229, and *p* = 0.761).

**Table 2:** Pairwise comparisons of groups based on depth of cavity

Group 1	Group 2	*p*-value	Cramér’s V
The layered model + VR	The layered model	0.524	0.274
The layered model + VR	VR	0.229	0.398
The layered model + VR	Control Group	< 0.001*	0.858
The layered model	VR	0.761	0.182
The layered model	Control Group	0.001*	0.694
VR	Control Group	0.019*	0.562

All p-values are based on Fisher-Freeman-Halton test

* indicates statistically significant results (*p* < 0.05)

The groups were compared in terms of overall cavity preparation quality, and no statistically significant difference was found among them (F(3,44) = 0.455; *p* = 0.715).

## Discussion

The integration of virtual reality (VR) and haptic simulators into dental education has been a subject of growing interest, aiming to enhance the acquisition of psychomotor skills essential for clinical proficiency. Our study evaluated the effectiveness of combined VR and the layered model training compared to traditional methods in occlusal amalgam cavity preparation using extracted human teeth [14]. The use of extracted teeth in preclinical training enables students to experience the tactile and anatomical characteristics of real dentition, providing a more realistic environment for assessing cavity preparation quality [15]. In our study, students who trained with both the layered model and VR demonstrated superior depth control compared to those who trained with extracted teeth alone, highlighting the effectiveness of these technologies in early manual skill development.

The results demonstrated a statistically significant improvement in the depth of cavity preparation among students who underwent VR and the layered model training, aligning with findings from previous studies that highlight the efficacy of VR-based simulations in dental education. A systematic review reported that VR simulation training significantly improved student performance and satisfaction compared to traditional methods [16]. Moreover, learners trained using haptic VR simulators demonstrated improved hand–eye coordination and tactile control during dental procedures. For instance, a systematic review by Moussa et al. concluded that virtual technologies positively influence dental education outcomes, particularly in skill acquisition and student satisfaction [17]. Similar outcomes were observed by Gandolfi et al., who emphasized the positive impact of haptic training tools in the development of fine motor skills among dental students [18]. Another recent systematic review highlighted that VR and interactive simulators significantly improve educational outcomes in dental training, suggesting these tools are well accepted by students and effective for psychomotor development [19].

Haptic feedback, which provides tactile sensations during simulation, plays a crucial role in developing manual dexterity and hand-eye coordination. Studies have shown that haptic simulators significantly enhance motor skill acquisition in preclinical dental training. This tactile interaction allows students to experience realistic resistance and textures, closely mimicking actual clinical scenarios [10, 16, 20]. For example, Zafar et al. demonstrated that students using haptic devices showed better tactile recognition and consistency in cavity depth [21].

Moreover, VR-haptic simulations offer individualized feedback and allow for repeated practice without the ethical and practical constraints associated with patient-based training. This flexibility has been shown to increase student engagement and confidence, facilitating a smoother transition to clinical practice [22–24]. Consistent with this, students in our The layered model + VR group and VR-only group achieved significantly better cavity depth scores compared to the control group (*p* < 0.001 and *p* = 0.019, respectively), supporting the role of VR in promoting critical tactile skills. Yang et al. noted improved learner motivation and satisfaction when VR was integrated with feedback systems [25]. Additionally, studies show VR-based assessments may contribute to more objective skill evaluation compared to traditional faculty grading [26].

Consistent with the findings of our study, Murbay et al. reported that undergraduate dental students trained with a virtual reality simulator achieved significantly higher rates of satisfactory cavity preparations than those who did not receive VR training. This suggests that the immersive and tactile feedback provided by VR simulators may facilitate better psychomotor skill development and spatial perception, particularly in depth-sensitive tasks such as occlusal cavity preparation [8].

Caviprep, a layered physical dental training model, offers a realistic and structured experience by mimicking the enamel, dentin, and pulp through materials of varying hardness. In line with this, students trained solely with the layered model also demonstrated significantly better depth control than the control group (*p* = 0.001), emphasizing the importance of tactile feedback even without VR. As students reach the red-colored pulp layer, they receive strong tactile feedback, enhancing their anatomical awareness and clinical sensitivity during cavity preparation. While limited studies have directly evaluated Caviprep, the principles behind layered base training models have been widely validated. For instance, the Learn-A-Prep II system has shown effectiveness in improving student hand skills and cavity preparation performance through its stratified structure [27]. Similarly, multi-layered caries models and VR-based layered digital teeth have been shown to significantly enhance psychomotor development and procedural accuracy in dental students [28, 29]. These models contribute to improved tactile perception and allow for more realistic practice of caries removal and depth control, which is crucial in occlusal cavity preparation.

Although both the layered model and VR training individually improved depth control compared to conventional methods, combining the two did not result in a statistically significant additive effect. This suggests that each modality independently supports skill acquisition, and their combined use may not necessarily lead to further improvement within a short training period. One possible explanation for the lack of additive effect is cognitive overload. According to Cognitive Load Theory, exposing novice learners to multiple complex training modalities—such as VR and layered models—simultaneously may exceed their working memory capacity, thereby impairing learning efficiency. This effect is particularly relevant in compressed training formats like ours, where students had limited time to adapt to each modality. Similar findings have been reported in simulation-based education literature, where increased cognitive load negatively impacted skill acquisition and transfer [30, 31]. Although no statistically significant differences were found in the overall cavity preparation scores (Table 3), this cumulative outcome serves as a useful summary indicator of training impact and complements the parameter-specific results [4, 5]. The absence of statistically significant differences in the overall cavity preparation scores (Table 3) may reflect the prioritization of foundational psychomotor skills-such as depth control-during the early phases of training. In contrast, more complex parameters like retention form or internal line angle likely require longer and more sustained practice periods to show measurable improvements.

**Table 3:** The comparison of four groups by overall cavity Preparation quality

Group	Mean ± SD	Median	Min-Max	*p*-value*	η2
The layered model + VR	6.75 ± 2.63	7.50	2.00–11.00	0.715	0.030
The layered model	7.08 ± 2.35	7.50	3.00–10.00		
VR	6.42 ± 2.07	7.00	3.00–9.00		
Control group	6.08 ± 1.68	6.50	2.00–8.00		

Mean ± SD: Mean ± Standard Deviation. η2: Eta-squared measure

* One-way ANOVA test result

Although we hypothesized that the combined use of VR and layered models would yield the greatest improvement in cavity preparation quality, this was not fully supported by the results. Only depth control showed significant enhancement in the simulation groups, and the overall performance scores did not differ significantly among groups. These findings suggest that while simulation tools offer specific benefits, their additive effect may be limited under short-term training conditions.

The significant difference observed only in the “depth of cavity” parameter may be due to the prioritization of depth control in early dental training, as it is a foundational skill that is heavily emphasized in preclinical curricula. Additionally, both the VR haptic system and the layered model are designed to enhance tactile and visual feedback, particularly related to depth, making it more responsive to simulation-based training. In contrast, parameters such as outline, marginal ridge integrity, and retention form may require more advanced spatial perception and manual refinement that develop over longer training periods or with more complex feedback systems.

The lack of an additive effect in Group 1 may be explained by several factors. First, the feedback mechanisms of the VR and layered model may overlap, resulting in redundant input rather than synergistic enhancement. Second, the relatively short training time may not have been sufficient for students to fully engage with and integrate both methods. Finally, from a cognitive load perspective, exposure to multiple novel technologies simultaneously may overwhelm novice learners and hinder effective learning. Future studies should investigate optimal sequencing or spacing of dual-modality simulation to avoid overload and maximize learning gains.

Despite improvements in depth control, no significant differences were observed in other cavity preparation parameters among the groups, including cavity outline, floor smoothness, internal line angles, marginal ridge integrity, retention form, and cavity width. This indicates that short-term simulation training may primarily enhance depth perception rather than overall cavity preparation quality.

Furthermore, the poor performance of the control group highlights the limitations of relying solely on traditional extracted tooth practice without simulation support. These findings underscore the importance of integrating advanced simulation technologies into preclinical training curricula to better prepare students for clinical practice.

However, the implementation of VR and haptic technologies in dental curricula is not without challenges. High costs associated with acquiring and maintaining advanced simulators can be a barrier for some institutions. Additionally, the effectiveness of these technologies depends on their integration into existing curricula and the quality of the simulations provided [23, 32]. Furthermore, as pointed out by Serrano et al., inadequate faculty training in simulator use can hinder effective adoption [23].

However, this study had certain limitations that must be considered. One important limitation was the relatively short duration of the haptic simulator training sessions. The limited training time might have contributed to the absence of significant differences among The layered model, VR, and The layered model + VR groups in parameters such as cavity outline, floor smoothness, internal line angles, marginal ridge integrity, and retention form, despite the significant improvement in depth control. Previous research has emphasized that adequate training time is crucial for students to fully benefit from haptic and VR technologies [22]​. The restricted exposure in our study may have limited the students’ ability to adapt to the tactile feedback and learning opportunities provided by the VR environment. This could partly explain the absence of significant differences among the layered model, VR, and The layered model + VR groups in parameters other than cavity depth. Future studies should consider extending the training period to better capture the potential advantages of these simulation tools [23]​. Moreover, the relatively small sample size and the inherent variability in the morphology and hardness of extracted teeth used for assessments may have introduced additional bias [15]​.

Our findings align with previous studies that support the effectiveness of VR-based simulators like Simodont and VirTeaSy in improving manual skills and depth perception during preclinical training [5]. However, unlike Simodont, which provides a fixed haptic interface, VirTeaSy allows some flexibility in simulating various anatomical scenarios [8]. The VR system used in this study provided depth feedback and resistance simulation but lacked contextual realism, which may explain the limited transfer effect on more complex preparation criteria. In contrast, the layered model (Caviprep) offers a physical, tactile experience that closely resembles real tissue stratification, making it particularly valuable for teaching fine motor control. Compared to 3D-printed teeth, which offer high standardization but often lack internal layering, Caviprep provides a structured progression through enamel, dentin, and pulp. Regarding practical implications, layered models are more cost-effective and logistically feasible for repeated group use, whereas VR simulators require substantial initial investment and technical maintenance [4]. Therefore, institutions with limited budgets may prioritize layered models as a more accessible yet pedagogically valid alternative, while VR could be selectively used for advanced individualized training.

Additionally, it must be acknowledged that certain device-specific features of the VirTeaSy system—such as enhanced visualization, rotation of the virtual model, and tactile feedback—may have influenced the depth perception outcomes observed in the VR group. Previous studies using platforms like Simodont have also demonstrated that such system functionalities can affect performance evaluation [33]. Furthermore, individual differences in stereoscopic vision among both students and evaluators may have contributed to variability in performance and scoring. These factors are now recognized as potential limitations of the current study, and future research should aim to isolate and evaluate the specific impact of environmental and user-related variables through more controlled experimental designs.

Another limitation is the unequal practice exposure among groups, as Groups 1, 2, and 3 received additional simulation training beyond the standard protocol provided to the control group. This introduces a potential dose effect, where increased practice opportunities could contribute to improved performance independently of the training modality itself. Therefore, the additional simulation time received by Groups 1–3 should be considered a potential confounding factor that may have influenced performance outcomes. To minimize this bias, future studies should aim to equalize total hands-on training time across groups when comparing instructional strategies.

Previous research has emphasized that adequate training time is crucial for students to fully benefit from haptic and VR technologies. Studies suggest that repeated simulation exposure over a period of 3 to 4 weeks, with at least 3 to 5 sessions, is often necessary to achieve measurable improvements in psychomotor skills and knowledge retention among novice dental students [4, 5]. In contrast, the present study utilized a relatively short training duration of two consecutive days, which may have limited the full potential impact of the simulation tools—particularly for complex parameters such as retention form or outline accuracy. This compressed timeframe was selected to reflect realistic scheduling constraints in preclinical curricula; however, the absence of significant differences in overall performance across groups may in part be attributed to this limited exposure. Future studies should therefore explore extended training timelines to determine the optimal frequency and spacing of VR and layered model sessions for sustainable skill development.

While this study adhered to simulation-specific reporting guidelines, some CONSORT criteria such as cost-effectiveness and long-term skill retention could not be fully addressed due to the short study duration and institutional constraints [11].

Another limitation of this study is the use of a compressed two-day training schedule, which may have restricted the acquisition of more complex manual skills. This timeframe was selected to reflect the constraints of preclinical curricula; however, longer protocols may better capture the full potential of simulation tools.

Another limitation lies in the use of extracted human teeth, which may vary in hardness, anatomy, and morphology, potentially introducing inter-group differences. Although preselection criteria were applied (e.g., exclusion of carious or restored teeth), full standardization is inherently challenging with biological specimens. As highlighted by Decurcio et al. [15], extracted natural teeth exhibit considerable variability in dentin and enamel hardness, which can influence training and assessment outcomes even when strict inclusion criteria are applied. Employing typodont or 3D-printed standardized teeth in future studies may further enhance methodological consistency.

Despite these challenges, the potential benefits of VR and haptic simulators, including tools like the layered model and other layered base training models, in dental education are substantial. They provide a safe and controlled environment for students to develop essential skills, reduce the risk of errors in real clinical settings, and can be tailored to individual learning paces. As technology advances and becomes more accessible, it is anticipated that these tools will become integral components of dental training programs [34–36].

While this study primarily focused on manual skill development, future research should consider incorporating secondary outcomes such as student engagement, usability, perceived realism, and long-term knowledge retention to better understand the broader educational impact of VR and layered simulation tools.

## Conclusion

This study demonstrated that the integration of virtual reality (VR) haptic simulators and layered physical models such as Caviprep improved cavity depth control among preclinical dental students. Students who received combined training with VR and the layered model achieved superior performance in cavity depth preparation compared to those trained with conventional methods alone.

## Electronic supplementary material

Below is the link to the electronic supplementary material.

Supplementary Material 1

## Data Availability

The datasets used and analyzed during the current study are available from the corresponding author on reasonable request.
