# Supplementary material for: Effects of virtual reality and layered tooth model training on manual dexterity in preclinical dental education
Source: BMC Med Educ. 2025 Jul 7;25:1020. doi: 10.1186/s12909-025-07622-9 (PMC12236048; doi:10.1186/s12909-025-07622-9)
Supplement: Supplementary file 1 — Supplementary Material 1 [file 12909_2025_7622_MOESM1_ESM.docx]

| **Category** | **Score 2** | **Score 1** | **Score 0** |
| --- | --- | --- | --- |
| Cavity Outline | Inclusion of all fissures; Not extending > 1/2 of cuspal incline | Extending > 1/2 but < 2/3 of cuspal incline | Extending > 2/3 of cuspal incline |
| Pulpal Floor |  | Flat pulpal floor | Pulpal floor is not flat |
| Internal Line Angles |  | Rounded internal line angles | Sharp internal line angles |
| Depth of Cavity | 1.5–2 mm | ± 0.5 mm deviation from ideal depth | > 0.5 mm deviation from ideal depth |
| Marginal Ridge | 2 mm | ± 0.5 mm deviation from ideal dimensions | > 0.5 mm deviation from ideal dimensions |
| Wall Direction (Facial & Lingual) | Convergent | Parallel | Divergent |
| Cavity Width |  | 1/4th - 1/5th intercuspal distance | Deviation from normal dimensions |

Supplementary Table 1 Class I Cavity Preparation Scoring Rubric

Evaluation criteria adapted from: Mittal P et al., BMC Oral Health. 2024;24(1):593.
